# In vitro chondrogenesis of Wharton’s jelly mesenchymal stem cells in hyaluronic acid-based hydrogels

**DOI:** 10.1186/s11658-016-0016-y

**Published:** 2016-08-12

**Authors:** Ewelina Aleksander-Konert, Piotr Paduszyński, Alicja Zajdel, Zofia Dzierżewicz, Adam Wilczok

**Affiliations:** 1 Department of Biopharmacy, School of Pharmacy with the Division of Laboratory Medicine in Sosnowiec, Medical University of Silesia, ul. Jednosci 8, 41-200 Sosnowiec, Poland; 2 Department of Health Care, Silesian Medical College, ul. Mickiewicza 29, 40-085 Katowice, Poland

**Keywords:** WJ-MSCs, Hydrogels, Chondrogenesis, Chondrocytes, HyStem, HyStem-C

## Abstract

**Background:**

In this study, we evaluated the usefulness of two commercially available hyaluronic acid-based hydrogels, HyStem and HyStem-C, for the cultivation of Wharton’s jelly mesenchymal stem cells (WJ-MSCs) and their differentiation towards chondrocytes.

**Methods:**

The WJ-MSCs were isolated from umbilical cord Wharton’s jelly using the explant method and their immunophenotype was evaluated via flow cytometry analysis. According to the criteria established by the International Society for Cellular Therapy, they were true MSCs. We assessed the ability of the WJ-MSCs and chondrocytes to grow in three-dimensional hydrogels and their metabolic activity. Chondrogenesis of WJ-MSCs in the hydrogels was determined using alcian blue and safranin O staining and real-time PCR evaluation of gene expression in the extracellular matrixes: collagen type I, II, III and aggrecan.

**Results:**

Chondrocytes and WJ-MSCs cultured in the HyStem and HyStem-C hydrogels adopted spherical shapes, which are characteristic for encapsulated cells. The average viability of the WJ-MSCs and chondrocytes in the HyStem hydrogels was approximately 67 % when compared with the viability in 2D culture. Alcian blue and safranin O staining revealed intensive production of proteoglycans by the cells in the HyStem hydrogels. Increased expression of collagen type II and aggrecan in the WJ-MSCs cultured in the HyStem hydrogel in the presence of chondrogenic medium showed that under these conditions, the cells have a high capacity to differentiate towards chondrocytes. The relatively high viability of WJ-MSCs and chondrocytes in both HyStem hydrogels suggests the possibility of their use for chondrogenesis.

**Conlusions:**

The results indicate that WJ-MSCs have some degree of chondrogenic potential in HyStem and HyStem-C hydrogels, showing promise for the engineering of damaged articular cartilage.

## Background

Articular cartilage has a limited capacity to regenerate. Even minor changes or injures can lead to its gradual damage and osteoarthritis. Tissue engineering Tissue engineering has potential for the regeneration of cartilage. The technique, which is still in its experimental stages, is based on a combination of three main components: suitable cells capable of differentiating towards chondrocytes; a three-dimensional scaffold; and specific signal factors [1, 2].

When selecting an ideal cell candidate, the following requirements must be met: availability and ease of isolation; high proliferative potential; the ability to produce extracellular matrix components and structures characteristic for cartilage; immunocompatibility; and the ability to integrate with the surrounding microenvironment. Mesenchymal stem cells (MSCs) exhibit huge potential in cartilage engineering [1, 3, 4].

The Wharton’s jelly (WJ) of the umbilical cord is an increasingly popular source of MSCs. WJ-MSCs show several advantages over cells derived from the bone marrow or adipose tissue of adult donors: ease of access; high proliferative potential; greater plasticity; and low immunogenicity [5]. A distinctive feature of WJ-MSCs is their capacity to self*-*renew and differentiate into chondrocytes, adipocytes, osteoblasts, skeletal myocytes, cardiomyocytes, neurons and endothelial cells [6].

Three-dimensional scaffolds used in cartilage tissue regeneration most often take the form of hydrogels, sponges or fibrous meshes made of natural and synthetic materials [7]. Hydrogels are particularly attractive due to their ability to absorb large amounts of water, tunable physical properties, homogenous cell distribution, high permeability for nutrients and waste products of metabolism, and ability to allow good physical integration into the defect. Moreover, hydrogels have the ability to mimic the native extracellular matrix (ECM) [8]. Biomaterials such as hyaluronic acid (HA) and gelatin are natural components that can be used to prepare polymeric hydrogels for tissue engineering. In particular, HA seems to be an excellent starting material for hydrogels thanks to its biocompatibility, biodegradability and excellent gelling properties, [8–10]. Gelatin can promote cell adhesion, differentiation and proliferation of the cells and it can be combined with other scaffold-forming materials due to its inferior mechanical properties [11, 12].

The aim of this study was to evaluate the usefulness of two commercially available HA-based hydrogels, HyStem and HyStem-C, for the cultivation of WJ-MSCs and their differentiation towards chondrocytes.

## Methods

### Cell cultures

Mesenchymal stem cells were isolated from the Wharton’s jelly of a human umbilical cord using the explant method. The sample was obtained in accordance with the guidelines set out by the Bioethical Committee of Medical University of Silesia, Katowice, Poland (consent number: KNW-6501-29/08).

Cells were cultured in standard growth medium containing α-modified minimum essential medium Eagle with L-glutamine and sodium bicarbonate (α-MEM; Sigma-Aldrich) supplemented with 20 % fetal bovine serum (FBS; Life Technologies), streptomycin (100 μg/ml; Sigma Aldrich), penicillin (100 U/ml; Sigma Aldrich) and non-essential amino acid solution 100× (NEAA; Life Technologies). Cells from the third passage were trypsinized, centrifuged and re-suspended in the hydrogel solutions.

To examine the mesenchymal phenotype, cells were analyzed via flow cytometry using a Human MSC Analysis Kit (BD Biosciences) according to the manufacturer’s protocol. A BD FACSAria II flow cytometer with FACS Diva Software 6.1.2. was used for the analysis.

Human CC-2550 chondrocytes isolated from the cartilage of the knee (Lonza) were used for comparison with the generated chondrocytes. The cells were cultured in Clonetics CGM Single Quots (Lonza) medium in a humidified atmosphere at 37 °C with 5 % CO_2_. Cells from the third passage were trypsinized, centrifuged and re-suspended in the hydrogel solution.

### Preparation of the hydrogel cell scaffolds

HyStem hydrogels were used as scaffolds for the encapsulation and proliferation of WJ-MSCs and their differentiation into chondrocytes. HyStem and HyStem-C hydrogels were obtained from the ESI BIO-A division of BioTime, Inc. HyStem components include thiol-modified hyaluronan (HyStem), thiol-reactive PEGSSDA cross-linker, and degassed, deionized water (DG Water). HyStem-C is fully chemically defined and based on three biocompatible components: thiol-modified hyaluronan (HyStem), thiol-reactive cross-linker, PEGSSDA, and thiol-modified gelatin (Gelin-S). The WJ-MSCs and chondrocytes were encapsulated in hydrogels at a density of 15,000 and 30,000 cells per 100 μl according to the manufacturer’s protocol. After 20 min of gelation, the appropriate cell culture media (100 μl) were added to each cell–hydrogel construct (100 μl). All the hydrogel constructs were incubated at 37 °C in 5 % CO_2_. The culture medium was changed every 2–3 days.

### Dissolution of hydrogels

Sixty mM stock solution of N-acetyl-L-cysteine (250 μl, NAC; Sigma-Aldrich) was added to 100 μl of the cell–hydrogel constructs to dissociate the hydrogel network. After 1 h incubation, the liquid from the wells was removed, placed in conical tubes and centrifuged at 1000 rpm for 5 min. Then the liquid was aspirated off and the cells were processed as desired.

### Cell viability of encapsulated cells

After 24 h and 7 days of culture, the metabolic activity of the cells cultured in the hydrogels was evaluated using a resazurin-based in vitro toxicology assay kit (TOX8, Sigma-Aldrich) according to the manufacturer’s protocol. The TOX8 assay was performed by transferring a 10 % solution of the dye to the wells of a 96-well plate filled with cells recovered from the hydrogels and incubated from 2 to 4 h at 37 °C in 5 % CO_2_. After exposure to resazurin, the absorbance of the converted dye was measured at a wavelength of 600 nm with a multiwell plate reader (Hewlett Packard 8452).

Cell viability was assessed after 7 and 21 days of culture by introducing acridine orange to the cell–hydrogel constructs. The cell–hydrogel scaffolds were washed three times with PBS and fixed with 4 % paraformalydehyde for 30 min at 4 °C. In the next step, the constructs were washed with PBS, and a dye mixture of acridine orange (5 μg/ml) was added and incubated for 10 min. The constructs were then washed with PBS and allowed to dry. A mixture of glycerol and water was added and the viable cells in the hydrogels were imaged using an Olympus BX 60 fluorescence microscope.

### In vitro chondrogenesis of WJ-MSCs encapsulated in hydrogels

We induced chondrogenesis in cell–hydrogel constructs by adding PromoCell MSC Chondrogenic Differentiation Medium. All of the cultures were grown for 21 days, then histological analyses were performed and total RNA was isolated for real-time PCR analysis. The calibrators (controls) used to analyze the relative changes in gene expression were WJ-MSC–hydrogel constructs grown in a standard growth medium.

### Histological analysis

To assess the presence of glycosaminoglycans (GAGs), which are cartilage-specific matrix proteins, cell–hydrogel constructs that had been cultured for 21 days in chondrogenic medium were stained with alcian blue and safranin O. After removal of the culture medium, the cell-seeded hydrogels were fixed in 4 % paraformalydehyde for 30 min, then washed twice with PBS before the addition of 0.1 % stock solutions of alcian blue and safranin O. After 30 min incubation at room temperature, the dye solution was removed and the constructs were washed with distilled water. The staining results were recorded under a Nikon Eclipse TS100 inverted microscope.

### RNA isolation and real-time PCR analysis

Total RNA was isolated using a NucleoSpin RNA Kit (Macherey-Nagel) according to the manufacturer’s protocol. Total RNA was isolated from three constructs per group. The amount of extracted RNA was checked using a Quant-IT RiboGreen RNA Reagent Kit (Life Technologies). Fluorometric measurements allowed the determination of the starting amount for real-time PCR. To remove possible DNA contamination, the samples were treated with DNAse I solution. The sequence of primers that we used in real-time PCR to detect collagen type I (COL1A1), collagen type II (COL2A1), collagen type III (COL3A1), aggrecan (ACAN), and glyceralaldehyde-3-phosphate dehydrogenase (GAPDH) were as follows:

- COL1A1 F: 5′ CCACCAATCACCTGCGTACA 3′, R: 5′ CATCGCACAACACCTTGCC 3′
- COL2A1 F: 5′ TGCTGACGCTGCTCGTCGC 3′, R: 5′ TCGTCGCAGAGGACAGTCCCA 3′
- COL3A1 F: 5′ CAGCAGGGTGCAATCGGCAGT 3′, R: 5′ TGGTTGCCCTGGGTGGCCT 3′
- ACAN F: 5′ CAAGAGCAGTGCAATCGTTGG 3′, R: 5′ ACATTCAGCTGCGGTTCCG 3′
- GAPDH F: 5′ GAAGGTGAAGGTCGGAGTC 3′, R: 5′ GAAGATGGTGATGGGATTTC 3′

GAPDH was used as a reference gene. Real-time PCR was performed in one step using a Power SYBR Green RNA-to-Ct-1-Step Kit (Applied Biosystems) under the following conditions: an initial denaturation (95 °C for 5 min) and polymerase activation (95 °C for 10 min) followed by 39 cycles of denaturation (95 °C for 15 s), annealing (60 °C for 30 s), and extension (72 °C for 30 s). To verify the reliability of the results obtained, each sample was analyzed in triplicate. The specificity and identity of the PCR products were checked using melting curve analysis. The relative gene expression was calculated using the comparative threshold cycle (ΔΔC_T_) method, where the fold difference was calculated using the expression 2^ΔΔCt^. REST 2009 software (Qiagen) was used to determine gene expression profiles and perform the statistical analysis.

### Statistical analysis

The results of the evaluations of the cell viability of encapsulated cells in hydrogels were checked for normality using the Shapiro-Wilk test and homogeneity of variance using Levene’s test. The results are shown as means and standard deviations (*n* = 6). The statistical differences between groups were calculated using one-way analysis of variance (ANOVA). To determine the precise differences between the means of the individual groups post-hoc RIR–Tukey’s test for unequal groups was used, performed with StatSoft’s STATISTICA version 10. Relative gene expression analyses were performed using the REST 2009 software (Qiagen). The significance level was set as *p* <0.05.

## Results

### WJ-MSC culture and phenotype characterization

The WJ-MSCs were able to adhere to the surface of the culture vessel and proliferate under standard culture conditions. A spindle-like shape with a clearly visible nucleus characterized the isolated WJ-MSCs. Flow cytometry showed that they expressed MSC-specific markers corresponding to those laid out in the criteria established by the International Society for Cellular Therapy. Cells were positive for CD90, CD105 and CD73, and negative for CD34, CD11b, CD19, CD45 and HLA-DR.

### Chondrocyte and WJ-MSC cultures in hydrogels

**Fig. 1:**
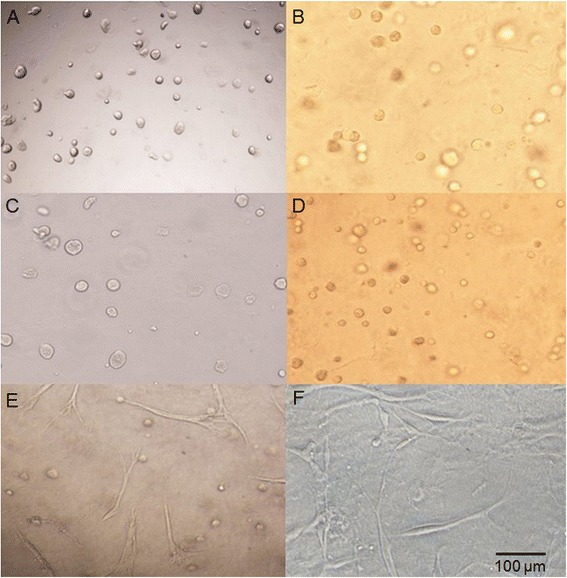
Cells cultured in hydrogels. **a** WJ-MSCs encapsulated in HyStem hydrogel. **b** Chondrocytes encapsulated in HyStem hydrogel. **c** WJ-MSCs encapsulated in HyStem-C hydrogel. **d** Chondrocytes encapsulated in HyStem-C hydrogel. **e** Attachment and spreading of WJ-MSCs on the surface of HyStem-C hydrogel. **f** Attachment and spreading of chondrocytes on the surface of HyStem-C hydrogel. Images taken using a Nikon Eclipse TS100 inverted microscope at a magnification of 200×; the scale bar is 100 μm

Chondrocytes and WJ-MSCs were spread well in the HyStem and HyStem-C hydrogels. Inverted light microscopy of cell-laden hydrogels revealed that the encapsulated cells were homogenously dispersed throughout the two hydrogels (Fig. 1). WJ-MSCs and chondrocytes adopted a characteristic round shape during the culture. The HyStem-C hydrogel increased attachment and spreading of cells on its surface, but this did not occur with the HyStem hydrogel (Fig. 1e and f).

#### Cell viability of encapsulated cells

**Fig. 2:**
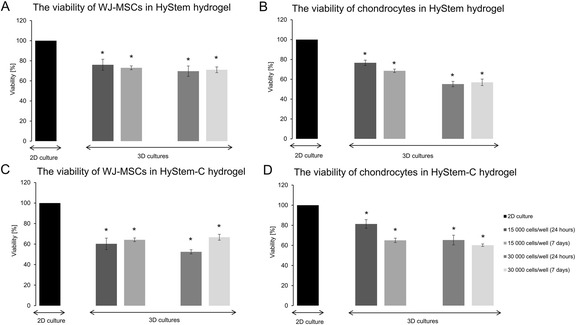
The viability of cells encapsulated in hydrogels. **a** The viability of WJ-MSCs in HyStem hydrogel after 24 h and 7 days of culture. **b** The viability of chondrocytes in HyStem hydrogel after 24 h and 7 days of culture. **c** The viability of WJ-MSCs in HyStem-C hydrogel after 24 h and 7 days of culture. **d** The viability of chondrocytes in HyStem-C hydrogel after 24 h and 7 days of culture. *p <0.05

Figure 2 shows the cell viability of encapsulated WJ-MSCs and chondrocytes in the HyStem and HyStem-C hydrogels after 24 h and after 7 days of culture in standard growth medium. The trend was a decrease in cell viability for all the constructs over 24 h and after 7 days compared to the viability in 2D cultures (defined as 100 %). In the case of WJ-MSCs encapsulated in the HyStem hydrogel at an initial density of 15,000 cells per well and 30,000 cells per well (Fig. 2a), the viability was similar and amounted to approximately 74.5 and 71 %, respectively. The viability of chondrocytes at an initial density of 15,000 cells per well in the HyStem hydrogel (Fig. 2b) showed similar values to the results obtained for the WJ-MSCs under identical culture conditions (Fig. 2a). Conversely, when the initial density of the chondrocytes in the HyStem hydrogel was 30,000 cells per well (Fig. 2b), a 20 % reduction in cell viability was observed compared to 15,000 WJ-MSCs per well and 30,000 WJ-MSCs per well (Fig. 2a) and 15,000 chondrocytes per well (Fig. 2b).

In the case of WJ-MSCs encapsulated in the HyStem-C hydrogel at an initial density of 15,000 cells per well (Fig. 2c), after 24 h and after 7 days of culture, the viability was similar and amounted to approximately 62 %. By contrast, the viability of WJ-MSCs at an initial density of 30,000 cells per well after 24 h of culture was 14 % lower than for the culture after 7 days (Fig. 2c). Fig. 2d shows that the viability of chondrocytes cultured in the HyStem-C hydrogel at an initial density of 15,000 cells per well after 24 h of culture was around 81.33 %, which was approximately 21 % higher than the WJ-MSCs (Fig. 2c). After 7 days of culture, the viability of chondrocytes at an initial density of 15,000 cells per well significantly decreased to a value of 64.86 % (Fig. 2d). When the initial density of chondrocytes was 30,000 cells per well, their average viability differed slightly in the cultures over 24 h and 7 days.

**Fig. 3:**
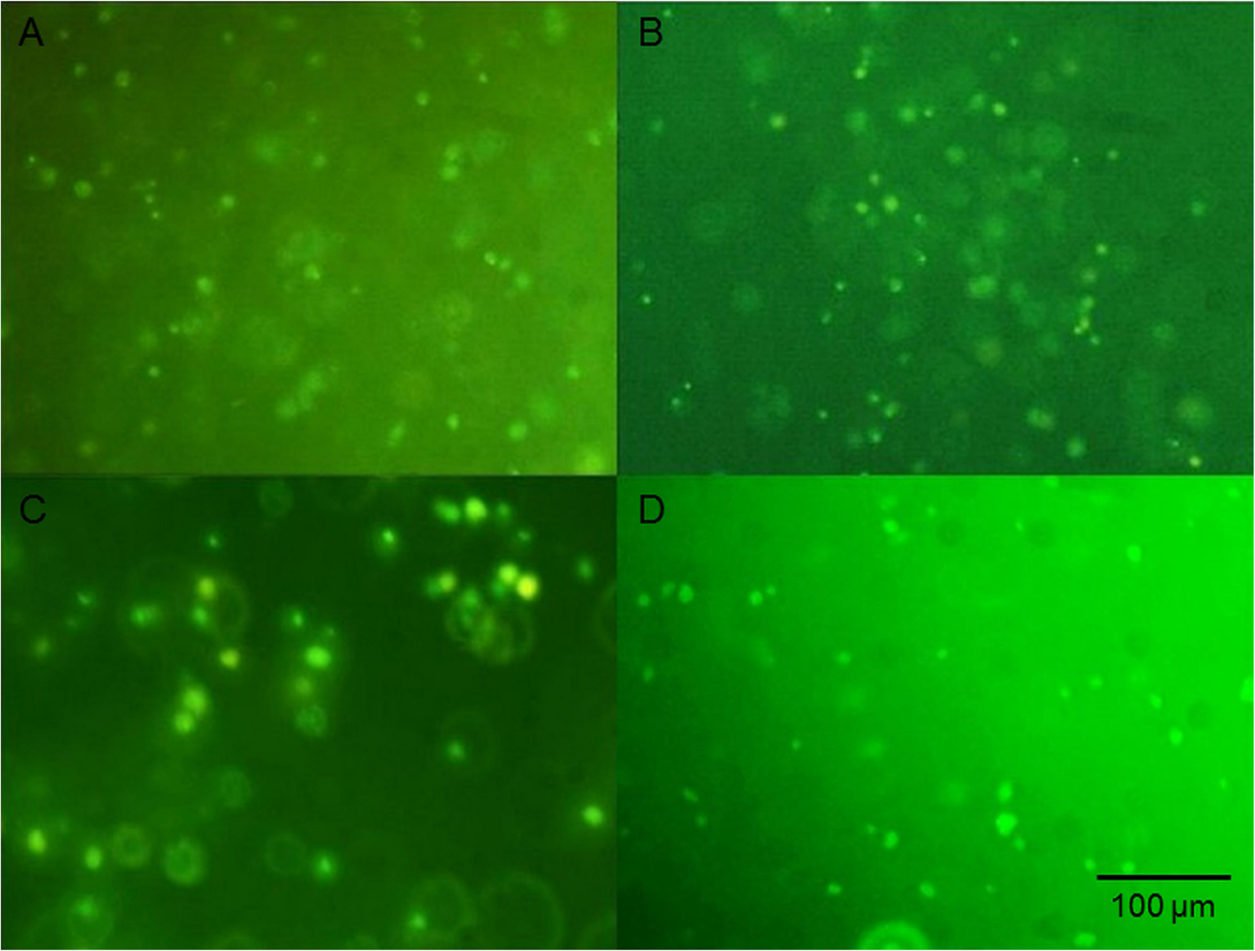
Viability staining of cells seeded in HyStem hydrogel. **a** WJ-MSCs cultured in HyStem hydrogel after 7 days of culture. **b** WJ-MSCs cultured in HyStem hydrogel after 21 days of culture. **c** Chondrocytes cultured in HyStem hydrogel after 7 days of culture. **d** Chondrocytes cultured in HyStem hydrogel after 21 days of culture. Images taken with an Olympus BX60 at magnification 200×; the scale bar is 100 μm

Fluorescence staining with acridine orange indicated that most of the cells remained viable throughout the hydrogels (Fig. 3). Green fluorescence of cells observed after staining confirmed that WJ-MSCs and chondrocytes survived in the 7- and 21-day cultures in the HyStem hydrogels.

### In vitro chondrogenesis of WJ-MSCs encapsulated in hydrogels

#### Histological analysis

**Fig. 4:**
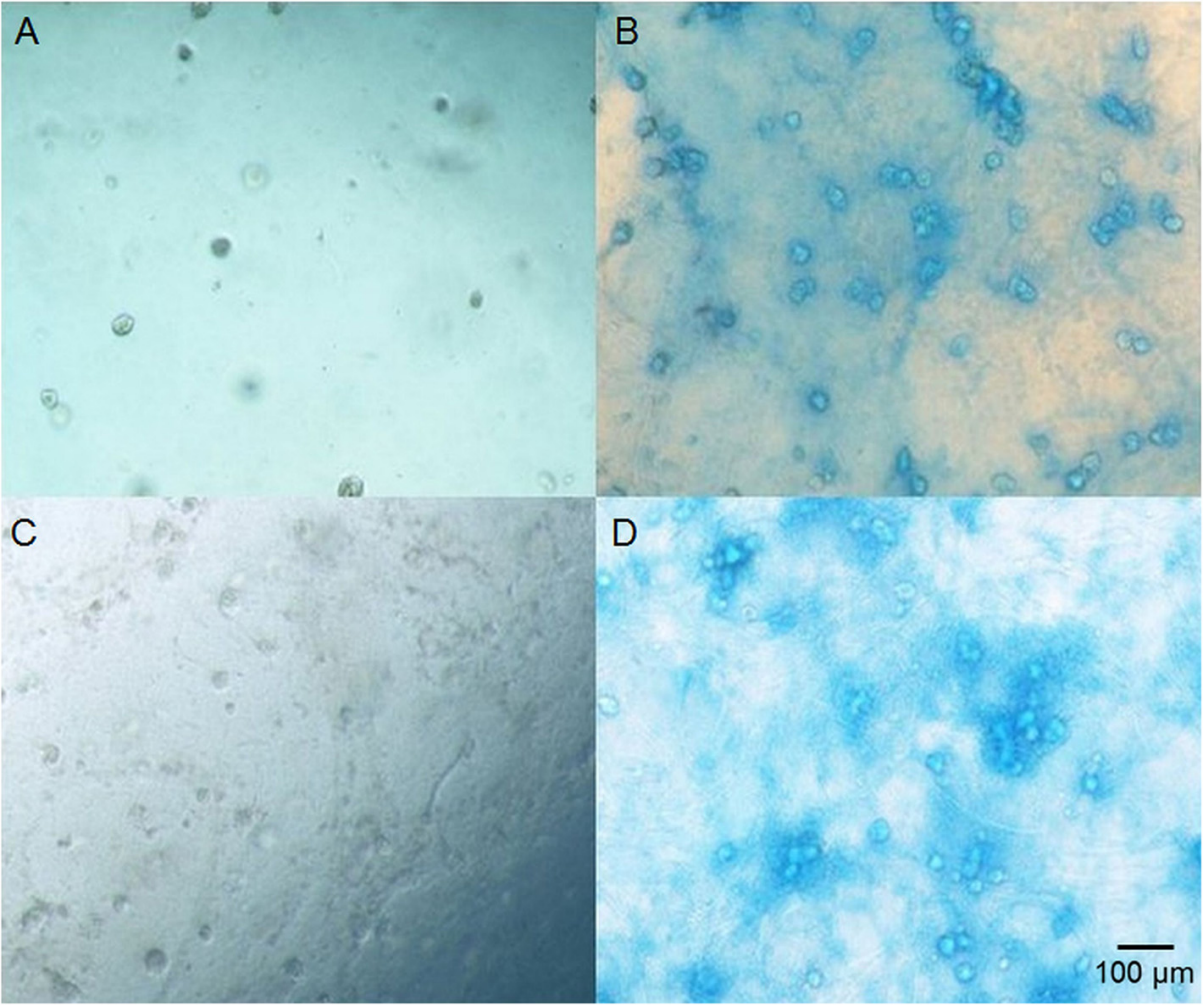
Alcian blue staining of GAG deposition in WJ-MSC-seeded hydrogels during chondrogenesis. **a** WJ-MSCs cultured in HyStem hydrogel in the presence of growth medium after 21 days of culture. **b** WJ-MSCs cultured in HyStem hydrogel in the presence of chondrogenic medium after 21 days of culture. **c** WJ-MSCs cultured in HyStem-C hydrogel in the presence of growth medium after 21 days of culture. **d** WJ-MSCs cultured in HyStem-C hydrogel in the presence of chondrogenic medium after 21 days of culture. Images taken using a Nikon Eclipse TS100 inverted microscope at a magnification of 100×; the scale bar is 100 μm

The accumulation of chondrogenesis-specific ECM in the hydrogel constructs was examined via histological staining, which revealed that the cells adopted a round morphology that is characteristic for chondrocytes. Alcian blue, which stains glycosaminoglycans (GAGs, cartilage-specific extracellular matrix), produced an intense blue color around the cells in the constructs after 21 days of culture in the presence of chondrogenic medium. This color was not observed in the cells cultured in standard growth medium (Fig. 4).

**Fig. 5:**
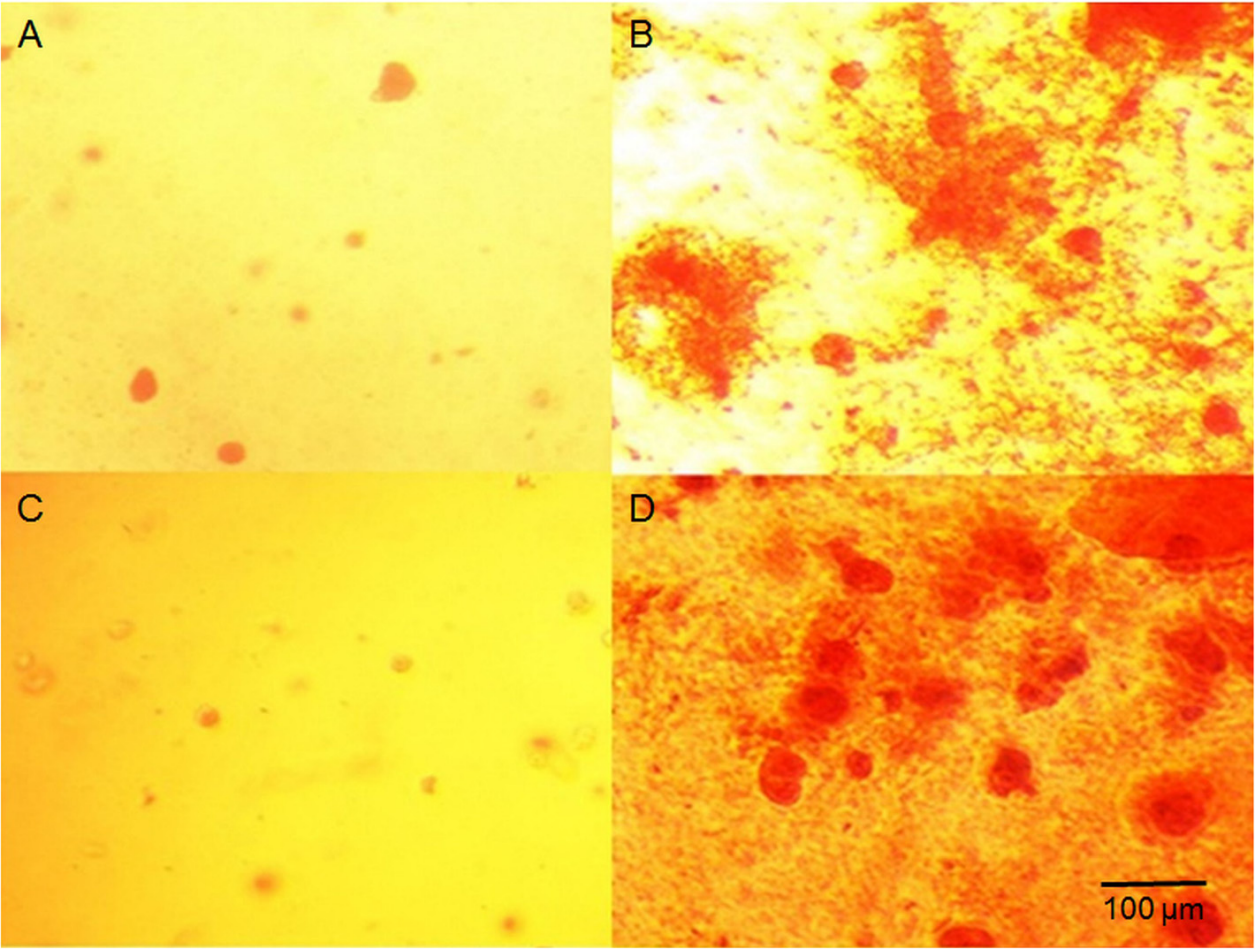
Safranin O staining of GAG deposition in WJ-MSC-seeded hydrogels during chondrogenesis. **a** WJ-MSCs cultured in HyStem hydrogel in the presence of growth medium after 21 days of culture. **b** WJ-MSCs cultured in HyStem hydrogel in the presence of chondrogenic medium after 21 days of culture. **c** WJ-MSCs cultured in HyStem-C hydrogel in the presence of growth medium after 21 days of culture. **d** WJ-MSCs cultured in HyStem-C hydrogel in the presence of chondrogenic medium after 21 days of culture. Images taken using a Nikon Eclipse TS100 inverted microscope at a magnification of 200×; the scale bar is 100 μm

The formation of GAGs was also confirmed via histological staining using safranin O dye, which produced an intense red–orange color in the constructs after 21 days of culture in chondrogenic medium compared to the cells with reduced ECM production, which were cultured without differentiating factors (Fig. 5).

#### Gene expressions

The expression of genes encoding matrix proteins produced by the cells encapsulated in the HyStem hydrogels was qualitatively detected using real-time PCR. After 21 days of culture, RNA was isolated and gene transcriptional activity was assessed for collagen types I, II and III and aggrecan. The reference against which the expression of particular genes was evaluated in the tested samples was 3D cultures of WJ-MSCs grown in the standard growth medium.

**Fig. 6:**
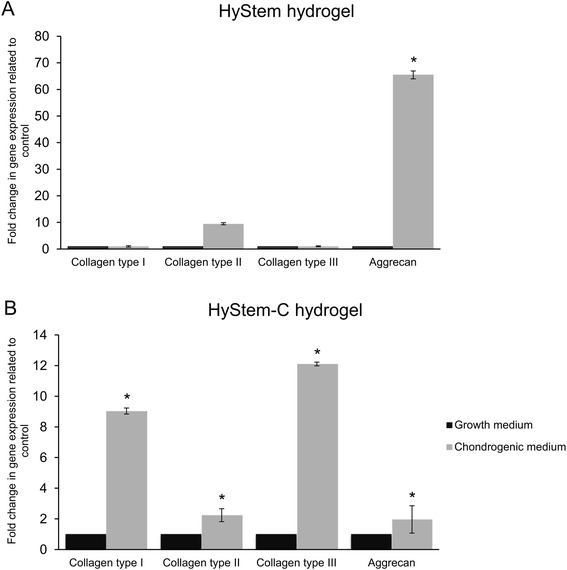
The transcriptional activity of genes (collagen type I, II and III and aggrecan) expressed as fold changes. **a** Expressions for WJ-MSCs cultured in HyStem hydrogel in the presence of growth medium after 21 days of culture compared to expressions for WJ-MSCs cultured in HyStem hydrogel in the presence of chondrogenic medium. **b** Expressions for WJ-MSCs cultured in HyStem-C hydrogel in the presence of growth medium after 21 days of culture compared to expressions for WJ-MSCs cultured in HyStem-C hydrogel in the presence of chondrogenic medium. *p <0.05

In the case of the HyStem hydrogel (Fig. 6a), the transcriptional activity of collagen type II gene, a key marker of chondrogenesis, increased significantly: approximately 9.5-fold in relation to the 3D culture in the standard growth medium used for the comparison. WJ-MSCs were also characterized by an approximately 65-fold increase in aggrecan expression in relation to the 3D culture in the standard growth medium. There were no significant changes in the expressions of collagen type I and III between the analyzed cultures.

As shown in Fig. 6b, WJ-MSCs encapsulated in the HyStem-C hydrogel were characterized by a marked increase in the expression of all of the analyzed genes compared to the expressions for those genes in the 3D culture without differentiating factors. We observed a 2.5-fold increase in collagen type II expression after 21 days of culture in the chondrogenic medium. The expression of aggrecan was approximately 12 times higher than in the corresponding cultures grown in standard growth medium. WJ-MSCs showed respective 9- and 2-fold increases in collagen type I and III transcriptional activity relative to the control culture of WJ-MSCs. Despite the concomitant increase in the expression of collagen type II and aggrecan, a significant increase in collagen type I and III was observed. It is noteworthy that the increase in collagen type II and aggrecan expression was not as high as in the case of the HyStem hydrogel cultures.

## Discussion

Current treatment methods for cartilage defects do not provide full regeneration of the damaged tissue. New cartilage regeneration systems could reduce the economic and social costs resulting from the need for treatment with conventional methods. 3D cell cultures show greater potential in regenerative medicine than 2D cultures. In this study, the chondrogenic differentiation of WJ-MSCs in HyStem and HyStem-C hydrogels was evaluated.

WJ-MSCs show exciting potential as a source of cells for the repair of damaged human articular cartilage. Their potential results from their ease of isolation without risk to the donor, low immunogenicity, ability to differentiate towards chondrocytes, capacity for self-renewal and low risk of transfer of bacterial or viral infection [13–15]. Flow cytometric analysis of isolated WJ-MSCs in this study showed high expression of human MSC markers and a lack of hematopoietic protein on the surface. Thus, umbilical cord Wharton’s jelly can be considered a unique, readily available source of early MSCs. WJ-MSCs are soon expected to become an alternative to MSCs derived from bone marrow.

The encapsulated cells were homogenously dispersed throughout HyStem and HyStem-C hydrogels. They adopted a characteristic round shape, which is an indicator of phenotype retention and is essential for matrix formation. Chondrocytes in native cartilage have a spherical shape and are surrounded by a cross-linked matrix that corresponds with the properties of the hydrogels [16]. Benya and Schaffer observed that the spherical shape of the cells is involved in maintaining the phenotype of chondrocytes. This manifests primarily through the synthesis of collagen type II and proteoglycans specific to the cartilage [17].

The viability of cells cultured in the hydrogels was evaluated using fluorescence staining and spectrophotometric assays determining the metabolic activity of living cells. The average viability of encapsulated WJ-MSCs in HyStem and HyStem-C hydrogels after 24 h and 7 days of culture was approximately 68 %, whereas the average viability of encapsulated chondrocytes was about 65 %. The relatively high viability of WJ-MSCs and chondrocytes in the HyStem hydrogels suggests the possibility of using them for chondrogenesis. The results obtained in this study allow us to conclude that the highly hydrated hydrogel could have a positive impact on the maintenance of cell viability due to adequate protection against mechanical stress and suitably high permeability to nutrients and metabolic products.

We evaluated the process of WJ-MSC chondrogenesis in HyStem and HyStem-C hydrogels. The results of histological staining with safranin O and alcian blue indicate that WJ-MSCs encapsulated in the hydrogel constructs with chondrogenic medium produced an abundant extracellular matrix that was rich in GAGs. It can be concluded that three-dimensional HA-based hydrogels help to retain the cellular phenotype of chondrocytes. Additionally, the expression of genes encoding ECM proteins produced by the cells encapsulated in the HyStem hydrogels during chondrogenesis was evaluated via real-time PCR. In the case of the HyStem hydrogel, a simultaneous increase in the expression of collagen type II and aggrecan was observed. This is highly desirable in the regeneration of cartilage structures, because collagen type II and aggrecan are major gene markers of hyaline cartilage [18, 19]. In addition, we observed a slight decrease in the expression of collagen types I and III, which are markers of fibrocartilage, which has an adverse effect in the regeneration of cartilage defects [20]. The upregulation of cartilage-specific genes in conjugation with the abundant production of cartilage ECM suggests high usefulness of the HyStem hydrogel to differentiate WJ-MSCs into chondrocytes. In the HyStem-C hydrogel, despite the simultaneous increase in collagen type II and aggrecan expression, we observed a significant increase in collagen type I and III expression.

Zhao et al. obtained similar results, demonstrating an increased transcriptional activity of collagen type I with a lesser increase in collagen type II and aggrecan expression after 14 and 21 days of culture in the presence of differentiation factors on 3D scaffolds made of PGA and PLLA. They suggested that in the presence of differentiation factors, a longer time is required for culture to enhance the expression of collagen type II and aggrecan [21]. Chen et al. also reported on the effect of time on MSC synthesis of collagen type II and aggrecan during chondrogenesis, noting that MSCs initially predominantly synthesized collagen type I and that the expression of collagen type II and aggrecan increased after a longer culture time [22]. Thus, the expression of collagen type I may be a marker of early chondrogenesis [23]. Changes in the composition and structure of the 3D scaffolds during culture can also affect the production of collagen type I in cells [24]. Increased expression of collagen type I in relation to collagen type II is characteristic for fibrous cartilage. Additionally, coexpression of collagen types I and II may indicate that the cells experienced a transition state between the characteristic phenotype of fibroblasts and chondrocytes [25].

It has also been reported that hypoxia or low oxygen tension may significantly influence the chondrogenesis of MSCs [26, 27]. Reppel et al. demonstrated an increase in chondrogenic differentiation when WJ-MSCs were expanded under conditions of hypoxia [28]. The HyStem-C hydrogel delivers signals to the interaction between the cell and the ECM components due to the informational signaling capacity, such as the RGD sequence. The results obtained in this study provide new information on the role of cell–ECM interactions in regulation of the process of chondrogenesis and indicate the importance of choosing the appropriate 3D scaffold for the treatment of damaged cartilage. Connelly et al. demonstrated the inhibitory effect of adhesion on the chondrogenesis of bone marrow-derived MSCs in 3D alginate gel modified with the RGD sequence, which caused the decreased expression of collagen type II, aggrecan and Sox-9 [29]. In the regeneration of articular cartilage, it is necessary to evaluate the effect of an artificially created environmental niche on the ability for self-renewal and differentiation of MSCs.

## Conclusions

The high viability of the isolated WJ-MSCs and chondrocytes in the HyStem hydrogels suggests the possibility of their use for chondrogenesis. Furthermore, the chondrogenesis of WJ-MSCs in the HyStem hydrogels yields cells with a phenotype similar to chondrocytes, which indicates the possibility of using these 3D scaffolds and cells in tissue engineering of damaged cartilage and may provide the basis for in vivo implantation.

## Abbreviations

ACAN, aggrecan; ADSCs, adipose mesenchymal stem cells; ANOVA, analysis of variance; BMSCs, bone marrow-derived stem cells; COL1A1, collagen type I; COL2A1, collagen type II; COL3A1, collagen type III; ECM, extracellular matrix; FBS, fetal bovine serum; GAGs, glycosaminoglicans; GAPDH, glyceralaldehyde-3-phosphate dehydrogenase; HA, hyaluronic acid; MSCs, mesenchymal stem cells; NAC, N-acetyl-L-cysteine; NEAA, non-essential amino acid solution; PBS, phosphate buffered saline; PGA, polyglycolic acid; PLLA, poly-L-lactide; TGF-β3, transforming growth factor β3; WJ-MSCs, Wharton’s jelly mesenchymal stem cells; α-MEM, α-modified minimum essential medium eagle
